# Supplementary material for: Dual-Modality Ultrasound Imaging of SPIONs Distribution via Combined Magnetomotive and Passive Cavitation Imaging
Source: Sensors (Basel). 2025 Nov 24;25(23):7171. doi: 10.3390/s25237171 (PMC12693940; doi:10.3390/s25237171)
Supplement: Supplementary file 1 [file sensors-25-07171-s001.zip › sensors-3937347-supplementary.pdf]

Magnetic Field Simulation

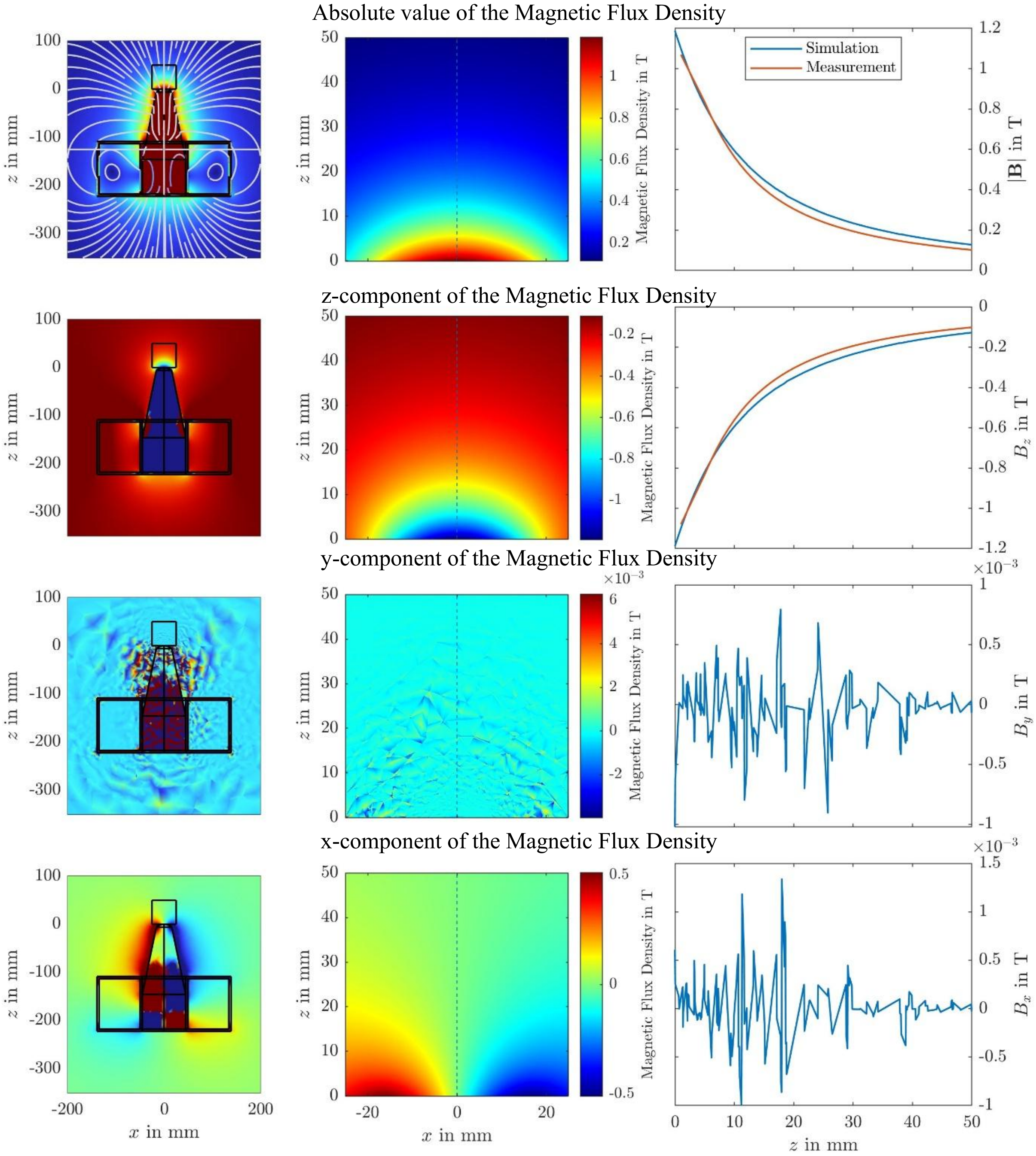

Figure. S1: Magnetic flux density simulation of the electromagnet in the xz-plane. The first column shows the entire electromagnet together with its surroundings. The second column provides a zoomed-in view of a 50 mm x 50 mm region directly above the pole tip. The third column presents the corresponding magnetic flux density values along a line from z=0 to 50 mm at x = 0 mm. Each row displays, in order, the absolute magnetic flux density followed by its z-, y-, and x-components.

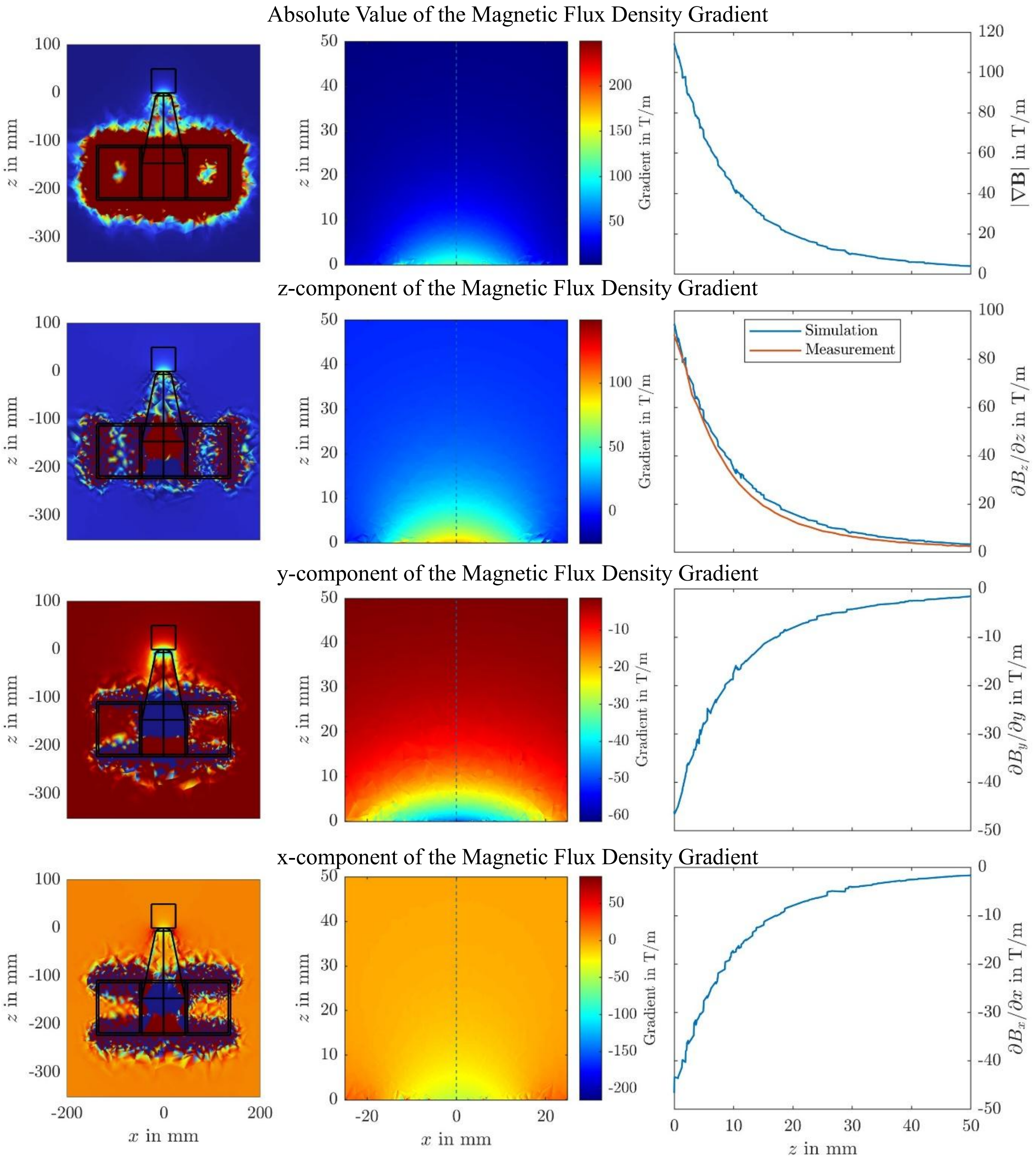

Figure. S2: Magnetic flux density gradient simulation of the electromagnet in the xz-plane. The first column shows the entire electromagnet together with its surroundings. The second column provides a zoomed-in view of a 50 mm x 50 mm region directly above the pole tip. The third column presents the corresponding magnetic flux density values along a line from z=0 to 50 mm at x = 0 mm. Each row displays, in order, the absolute magnetic flux density gradient followed by its z-, y-, and x-components.
